# Abstracts from Foreign Journals

**Published:** 1902-10

**Authors:** 


### ABSTRACTS FROM FOREIGN JOURNALS.

### FRENCH.

UNDER THE DIRECTION OF S. J. J. HARGER, V.M.D.,
PHILADELPHIA, PA.

CORROSIVE SUBLIMATE IN FOOT-AND-MOUTH DISEASE. Hirzel, after treating a herd of cattle infected with aphthous fever, came to the same conclusions previously made by others. The sublimate injection, intravenously or subcutaneously, did not prevent the disease in those animals exposed to the infection. Likewise, it did not have any influence upon the evolution of the disease when given after the animal had shown symptoms. Its effect was neither prophylactic nor curative. In some cases the injection produced mercurial intoxication in from ten to fourteen days. The most prominent symptoms consist of moist, exanthematous scales around the udder and vulva in the cow, and the base of the tail, mouth, head, and neck in younger animals. The animal generally makes a rapid recovery. Hirzel has also observed that a previous attack of foot-and-mouth disease gives a degree of immunity to the affected animal which within a certain limit is hereditary to the offspring. Much was only a short time ago claimed for Bocelli's treatment of foot-and-mouth disease, but nearly all observers who have employed this method are in accord that it deserves little or no merit.—*Ann. de Med. Vet.*

MECHANISM OF THE PATHOGENIC ACTION OF SOME HELMINTHS. The pathogenic rôle of certain nematodes has been contested, especially in gastro-intestinal strongylosis of sheep. The rôle of these parasites in the abomasum can be readily determined from the fact that after their expulsion the cachectic symptoms commence to disappear.

The sclerostoma of the horse, immediately after death, are found adherent to the mucosa, to which they are fixed by the sucking action of the mouth. In this manner they nourish themselves by drawing blood from the intestinal capillaries, and at these points leave small wounds of the mucous membrane visible to the naked eye, through which infection of intestinal origin may take place.

The tricocephala of the pig penetrates the mucous membrane for a distance of 1 to 1.5 centimetres.

The ascarides of the horse do not produce any alteration in the mucous membrane.—*Soc. des Sc. veter. de Lyon.*

Anomaly of the Kidneys. In an adult pig the two kidneys were united at their postero-inferior part by a commissural band of renal tissue 3 centimetres wide and 1.5 centimetres thick, giving them the resemblance of a horseshoe. Otherwise the organs were normal.—*Ann. de Med. Vet.*

Abscess of the Spleen Caused by Swallowing a Needle. Subject, colt, aged eighteen months; sick four days. Symptoms: Loss of appetite, chills, shallow, painful respirations, colicky pains, lying down in the left sterno-abdominal position. Percussion of chest normal on both sides; percussion over left hypochondriac region is painful. On the right side the vesicular murmur of the lungs was increased and on the left side completely absent. The cardiac sounds were strong and metallic. Bergeon suspected pleurisy. The next day the abdominal pain increased and the horse died.

Autopsy: Acute peritonitis, especially in the region of the stomach; liver resembling that seen in septic infection; spleen the seat of a tumor the size of a man's head adherent to the stomach and diaphragm. The tumor was a large abscess in which was found a needle 4.5 centimetres long. The passage of the needle, which the animal swallowed, through the stomach was indicated by a small cicatrix.—*Ann. de Med. Vet.*

Renal Colic with Albuminuria and Hæmaturia. Brun reports a horse, aged nine years, suffering from attacks of abdominal pain while in the stable. The symptoms were not diagnostic. Rectal exploration revealed nothing except a few drops of coffee-colored urine escaping from the urethra. The next day intestinal fermentation necessitated the use of the trocar. Examination of the urine showed that it contained a large percentage of albumin, some blood, pus, and epithelial débris. This fact showed that it was here a question of a nephritis which had been the cause of the several attacks of intestinal indigestion.

A second examination of the urine eight days afterward revealed no abnormal elements in this excretion, and up to that time the intestinal trouble had not recurred.

. . . Brun relates a second case, clinically almost identical with the first. He expresses the opinion that in all cases of abdominal disorders accompanied by pain which is not plainly attributable to indigestion, the examination of the urine should not be omitted. In fact, in nearly all internal diseases in which the pathology and diagnosis are not very evident, an examination of the urine should be made.—*Ibid.*

Toxin of the Tapeworm. The researches of Messineo and Calamida support the ancient theory of the injurious effects of tapeworms and some other intestinal helminths, caused by the elaboration of a toxin by them rather than by their mechanical irritation.

A watery extract of these parasites was injected under the skin into the peritoneal cavity and the brain of a large number of guinea-pigs, rabbits, and dogs. The symptoms in all cases were uniform: staring coat, trembling, general depression, paralysis of the hind-quarters, and lowering of the temperature.

Autopsy: Capillary dilatation of the liver with small hemorrhagic foci at the periphery of the hepatic lobules; granulo-fatty degeneration of the hepatic cells and infiltration of the perivascular connective tissue, with leucocytes. The kidneys showed fatty degeneration of the epithelial cells, capillary dilatation and hemorrhagic foci in the Malpighian glomeruli. Capillary dilatation in the brain, but no lesion of the nerve cells. The authors attribute to the tænia a special toxin of pathogenic action.—*Ibid.*

Exocardia. Schrader relates the following anomaly: A lamb presented underneath the sternum a round tumor, which, after examination, was recognized as the heart. Attempts at reduction threatened asphyxia. At the autopsy—eight days after birth—the enlargement was found to consist of the heart surrounded by the pericardium, which had descended through a round opening in the sternum. The pericardium was firmly attached to the opening in the sternum, which was at a point corresponding to the place where the apex rests in a normal state.—*Ibid.*

Remarks on the Sero-diagnosis of Glanders. The classical methods of experimental diagnosis—inoculation and malleination—are not always practicable and do not always furnish precise results. The sero-diagnostic method, therefore, deserves consideration.

This method of sero-diagnosis, based upon the *phenomena of agglutination*, has for some years been investigated by diverse experimenters in reference to the blood serum obtained from glandered horses. Recently Rabieaux, of the Lyons Veterinary School, has made known the result of his work carried on during the last two years in the laboratory of M. Galtier. These researches were confined to glandered horses (eight chronic, one acute) and eleven healthy horses.

The phenomena of agglutination of serums glandered or non-glandered vary in intensity and rapidity according to the temperature to which they have been previously exposed and the degree of dilution. Rabieaux found that the agglutination in cases of glanders manifests itself very intensely in a dilution of 1 : 1500, and takes place rapidly when the serum is obtained from a horse with a high temperature—a condition in which the result of malleination is unreliable.

An injection of mallein does not modify the agglutinating power of the glanders serum if it be abstracted after the temperature has again become normal. During the period of elevation of temperature this power is increased.

From his researches Rabieaux has formulated the following conclusions:

The difference between the agglutinating power of the serums obtained from the glandered and from the healthy horse is sufficiently decided that this property may serve as a means of experimental diagnosis of glanders.

An animal which furnishes a serum with an agglutinating power in a dilution of 1 : 1000 should be considered as suffering from glanders.

The method of the sero-diagnosis of glanders, because of its technique, belongs to the laboratory. Therefore, it should not entirely replace the experimental methods of diagnosis ordinarily employed, which are much more practicable and can be practised in almost any place. Nevertheless, conditions may arise in which the other methods fail or cannot be successfully carried out. In such instances the examination of the blood serum may be employed as a control method, or it may be the only condition upon which a diagnosis is possible. It is especially serviceable in making a diagnosis upon the cadaver, and as such is useful in the inspection of meat.

It is useful, when examining a specimen of suspected blood serum, to examine at the same time the serum from a healthy horse. —*Ibid.*

